# Esophageal cancer associated with a sarcoid-like reaction and systemic sarcoidosis in lymph nodes: supportive findings of [18F]-fluorodeoxyglucose positron emission tomography–computed tomography during neoadjuvant therapy

**DOI:** 10.1186/s40792-018-0473-9

**Published:** 2018-06-25

**Authors:** Takayoshi Kishino, Keiichi Okano, Yasuhisa Ando, Hironobu Suto, Eisuke Asano, Minoru Oshima, Masao Fujiwara, Hisashi Usuki, Hideki Kobara, Tsutomu Masaki, Emi Ibuki, Yoshio Kushida, Reiji Haba, Yasuyuki Suzuki

**Affiliations:** 1 Department of Gastroenterological Surgery, Kagawa University, 1750-1 Ikenobe, Miki-cho, Kita-gun, Kagawa, 761-0793 Japan; 2 Department of Gastroenterology and Neurology, Kagawa University, Kagawa, Japan; 3 Department of Diagnostic Pathology, Kagawa University, Kagawa, Japan

**Keywords:** Esophageal squamous cell carcinoma, Sarcoidosis, Sarcoid-like reaction, FDG-PET, Bilateral hilar lymphadenopathy

## Abstract

**Background:**

In patients with esophageal cancer, differentiation between lymph node metastasis and lymphadenopathies from sarcoidosis or sarcoid-like reactions of lymph nodes is clinically important. Herein, we report two esophageal cancer cases with lymph node involvement of sarcoid-like reaction or sarcoidosis.

**Case presentation:**

One patient received chemotherapy and the other chemoradiotherapy as initial treatments. In both cases, [^18^F]-fluorodeoxyglucose positron emission tomography–computed tomography (FDG-PET/CT) was performed before and after chemo(radio)therapy. After the treatment, FDG uptake was not detected in the primary tumor, but it was slightly reduced in the hilar and mediastinal lymph nodes in both cases. These non-identical responses to chemo(radio)therapy suggest the presence of sarcoid-like reaction of lymph nodes associated with squamous cell carcinoma of the esophagus. Curative surgical resection was performed as treatment.

**Conclusions:**

These FDG-PET/CT findings may be helpful to distinguish between metastasis and sarcoidosis-associated lymphadenopathy in esophageal cancer.

## Background

[^18^F]-fluorodeoxyglucose positron emission tomography–computed tomography (FDG-PET/CT) is a useful diagnostic tool [1, 2]. FDG-PET/CT is based on the metabolic state of cells and thus yields new information that is largely different from that of a computed tomography (CT) or magnetic resonance imaging (MRI) scan. FDG-PET/CT is also useful for monitoring the effects of chemotherapy and chemoradiotherapy [3–5]. However, it is still difficult to establish an accurate diagnosis in some situations. For example, FDG-PET/CT might detect highly integrated non-metastatic lesions that might be incorrectly diagnosed as metastases. Non-metastatic lesions with a high FDG uptake are often caused by sarcoidosis, sarcoid-like reactions, or an infection.

Sarcoidosis is a systemic inflammatory disease of an unknown cause that is characterized by the widespread development of non-caseating granulomas in more than one organ system, frequently hilar and mediastinal lymph nodes [6]. Sarcoid-like reactions are also observed as epithelioid cell granulomas of the lymph nodes due to a malignancy [6–11]. Although the coexistence of these lymphadenopathies with esophageal cancer is relatively uncommon, the ability to differentiate these cases from lymph node metastasis is crucial. Moreover, clinical staging based on an accurate diagnosis of these lymphadenopathies is important for selecting the most appropriate treatment.

Herein, we describe two cases of esophageal cancer in which FDG-PET/CT revealed high FDG accumulation in the hilar and mediastinal lymph nodes owing to sarcoidosis or sarcoid-like reactions.

## Case presentation

### Case 1

**Fig. 1:**
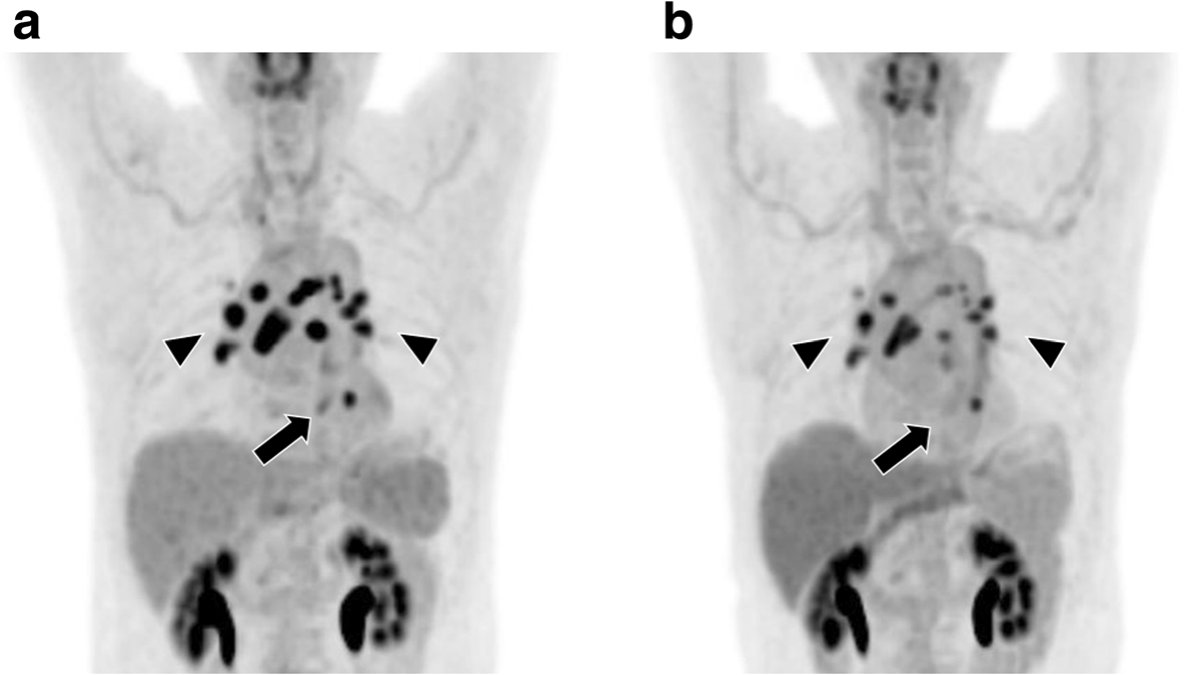
FDG-PET/CT images obtained before (**a**) and after (**b**) chemotherapy in case 1. **a** Images obtained before chemotherapy show FDG accumulation in the primary cancer (arrow; SUV max: 4.06) and in the hilar-mediastinal lymph nodes (arrow head; SUV max: 15.0). **b** Images obtained after chemotherapy show a disappearance in FDG uptake in the primary cancer (arrow) and a slight reduction in FDG uptake in the hilar-mediastinal lymph nodes (arrow head; SUV max: 8.07)

**Fig. 2:**
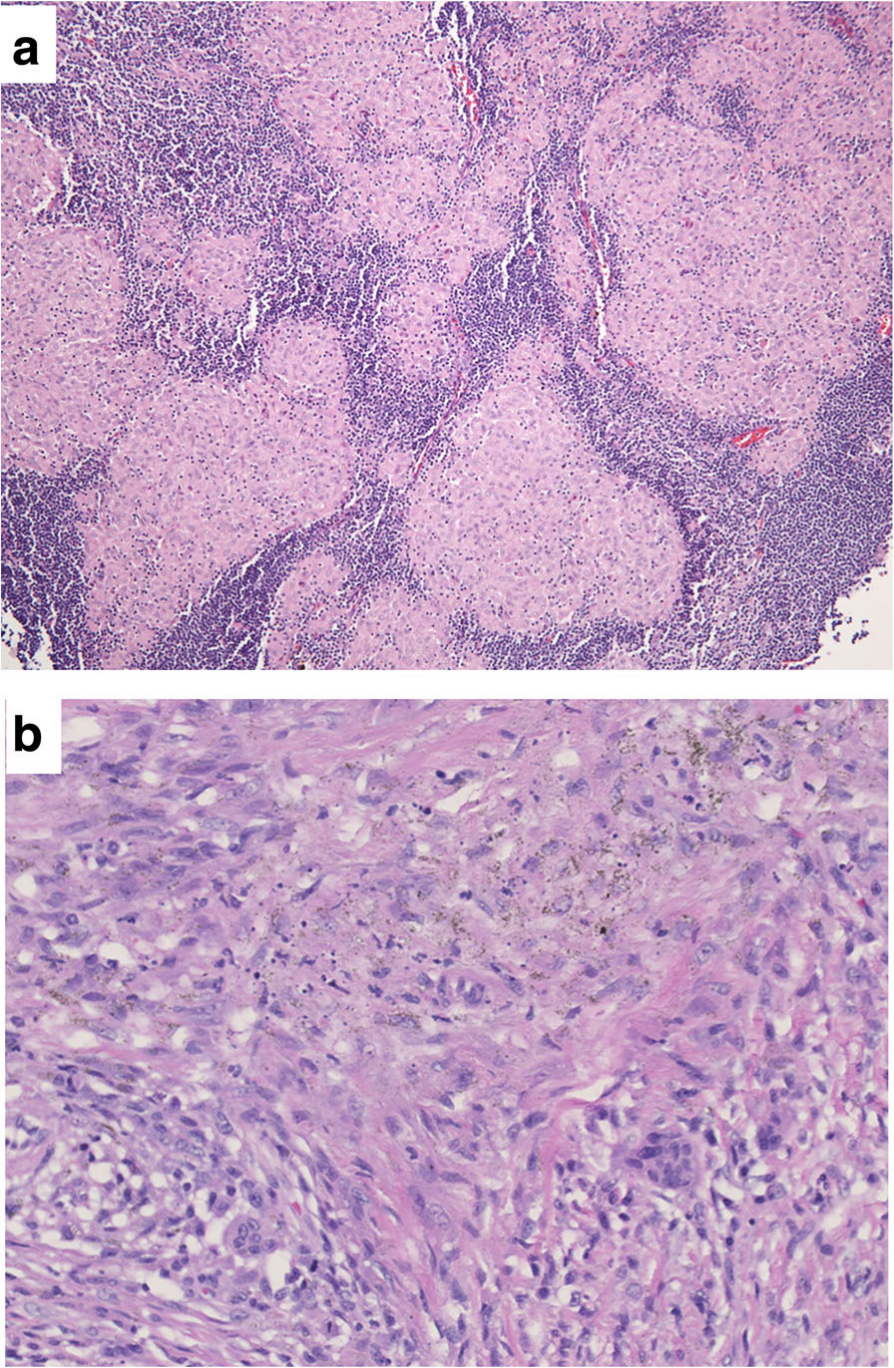
Histology of lymph nodes resected from case 1. A photomicrograph of the specimens shows granulomatous reactions, histiocytes, multinucleated giant cells, and scar-like fibrosis without malignant cells (**a** magnification × 100, **b** × 400, Hematoxylin and Eosin staining)

A 70-year-old man underwent an endoscopic examination owing to epigastric pain and was diagnosed with esophageal cancer. The endoscopic examination revealed an irregular mucosa in the lower esophagus, and biopsies confirmed squamous cell carcinoma. Contrast-enhanced CT did not depict the esophageal lesion but showed enlarged lymph nodes in the tracheal bifurcation and bilateral hilum of the lung. FDG-PET/CT revealed abnormal accumulation in the main tumor in the lower esophagus (maximum standardized uptake value [SUV max]: 4.06) and higher accumulation in the hilar-mediastinal lymph nodes (SUV max: 15.0) and enlarged mediastinum lymph nodes (SUV max: 6.94) (Fig. 1). The primary lesion of the esophagus was staged T1; nevertheless, it was still difficult to rule out metastasis in the lymph nodes. We selected chemotherapy as the first-line treatment. The patient was administered 2 cycles of 140 mg cisplatin and 1400 mg 5-fluorouracil over 2 months. In each cycle, 9.9 mg dexamethasone was administered to prevent side effects of chemotherapy. Subsequently, we observed a disappearance of the FDG uptake in the primary lesion, and a slightly reduced FDG uptake in the mediastinal and bilateral hilar lymph nodes (Fig. 1). These non-identical responses to chemotherapy did not indicate cancer metastasis, but most likely a sarcoid-like reaction of the lymph nodes associated with squamous cell carcinoma of the esophagus. Therefore, the patient underwent video-assisted thoracoscopic surgery esophagectomy (VATSE) with gastric tube reconstruction via the retrosternal route. The pathological diagnosis was moderately to poorly differentiated squamous cell carcinoma of the lower thoracic esophagus. The resected lymph nodes demonstrated no tumor metastasis. However, some lymph nodes (#8a, #106RecR, #107, #108, #109) showed granulomatous reactions, histiocytes, multinucleated giant cells, and scar-like fibrosis (Fig. 2), suggesting the presence of sarcoidosis or sarcoid-like reactions. In accordance with the Union for International Cancer Control (UICC) TNM staging system (7th edition), the tumor was classified as pT1N0M0, pStage IA. The patient was discharged 49 days after surgery. FDG-PET/CT performed 15 months after surgery showed bilateral FDG accumulation in the hilar lymph nodes without tumor recurrence.

### Case 2

**Fig. 3:**
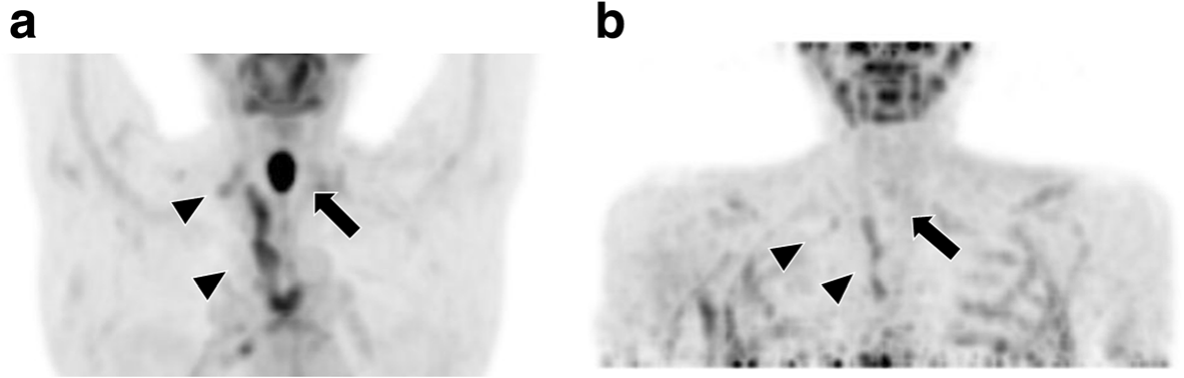
FDG-PET/CT images obtained before (**a**) and after (**b**) chemotherapy in case 2. **a** Images obtained before chemoradiotherapy show FDG accumulation in the primary cancer (arrow; SUV max: 23.1) and in the mediastinal lymph nodes (arrow head; SUV max: 5.45). **b** Images obtained after chemotherapy show a disappearance in FDG uptake in the primary cancer (arrow) and a slight reduction in FDG uptake in the mediastinal lymph nodes (arrow head; SUV max: 3.26)

**Fig. 4:**
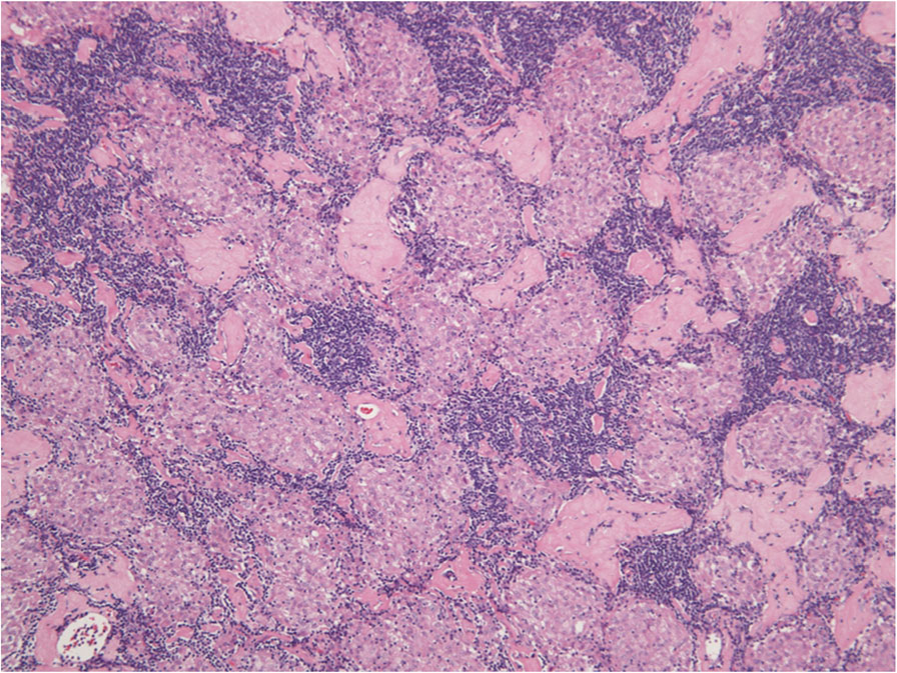
Histology of lymph nodes resected from case 2. A photomicrograph of the specimen shows granulomatous reactions containing several small epithelioid cell granulomas without malignant cells (magnification × 100, Hematoxylin and Eosin staining)

A 72-year-old woman with a past medical history of sarcoidosis underwent an endoscopic examination owing to dysphagia. The endoscopic examination revealed a circumferential tumor with ulceration in the cervical esophagus. Biopsies obtained during the endoscopy indicated squamous cell carcinoma. Contrast-enhanced CT showed extensive tumor growth with suspected tracheal invasion and enlarged lymph nodes extending from the cervical region to the upper mediastinum. FDG-PET/CT revealed abnormal FDG accumulation in the primary lesion (SUV max: 23.1) and lymph nodes (SUV max: 5.45) from the cervical to upper mediastinal region (Fig. 3). However, it was difficult to determine whether the multiple lymphadenopathy was benign or metastatic because of her past medical history of sarcoidosis. Therefore, and also for the purpose of preserving the larynx, we initiated definitive chemoradiotherapy. The patient was administered 2 cycles of 45 mg cisplatin and 700 mg 5-fluorouracil with 60 Gy/30 fr radiation therapy over 2 months. In each cycle, 8 mg dexamethasone was administered to prevent side effects of chemotherapy. After completing the chemoradiotherapy, we observed complete disappearance of FDG uptake in the primary cancer in the esophagus, and only a slight reduction in FDG uptake in the mediastinal lymph nodes (SUV max: 3.26; Fig. 3), which indicated that the lymph nodes were affected by sarcoidosis. The primary lesion of the esophagus relapsed 3 months later. Then, the patient underwent thoracoscopic and laparoscopic total laryngopharyngoesophagectomy with gastric tube reconstruction via the posterior mediastinal route. The pathological diagnosis was moderately differentiated squamous cell carcinoma in the cervical esophagus. The resected lymph nodes demonstrated no tumor metastasis. However, some lymph nodes showed granulomatous reactions and contained several small epithelioid cell granulomas (Fig. 4), suggesting the presence of sarcoidosis. The final stage was determined as pT2N0M0, pStage IB (UICC 7th). The patient was discharged 27 days after surgery. Contrast-enhanced CT performed 6 months after surgery showed no tumor recurrence. However, the patient died of myocardial infarction 1 year after surgery.

## Conclusions

Sarcoidosis or sarcoid reactions can occur in association with a malignancy [12]. Sarcoidosis is characterized by the formation of non-caseating granulomas in multiple organ systems and is associated with symptoms such as dyspnea, low-grade fever, weight loss, skin rashes, and vision changes [13]. Sarcoidosis is treated using glucocorticoids, immunosuppressants, and hydroxychloroquine. In contrast, sarcoid-like reactions lack the clinical symptoms of sarcoidosis [12]. The patient from case 1 in this report did not have symptoms or laboratory data suggestive of sarcoidosis, although the lymph nodes contained histiocytes, multinucleated giant cells, and scar-like fibrosis. Therefore, we diagnosed this patient with sarcoid-like reaction, which did not require further treatment.

The causal relationship between malignant tumors and sarcoid-like reaction in regional lymph nodes remains unclear. It is possible that sarcoid-like granulomas represent a T-cell-mediated immune reaction to an antigenic expression by the tumor [14]. Sarcoid-like reactions are not rare in patients with cancerous lesions, occurring in about 5% of patients [15, 16]. Therefore, it is very important to differentiate lymph node metastasis from sarcoid-like reactions, because incorrect diagnosis of lymphadenopathy may lead to inappropriate treatment. However, in fact, it is difficult to determine whether the lymph node lesion is a metastatic or benign lesion in patients with concurrent malignant tumors and lymphadenopathy [17]. Parra et al. suggested that the only way to differentiate these lesions is to perform biopsies and histopathological tests [17]. Lymphadenopathy, however, may include both metastasis and sarcoid reactions [18]. Bekki et al. suggested that a biopsy could provide potentially misleading information and recommended video-assisted thoracoscopic surgical lymph node dissection [11]. However, such dissection is an invasive technique. In addition, surgical interventions including VATS promote adhesion around lesions which may increase the difficulty of subsequent surgery. Bekki et al. also suggested that monitoring responses to chemotherapy with FDG-PET/CT was useful for diagnosing metastatic or benign lesions, in a patient with concurrent malignant tumors and lymphadenopathy [11].

We first administered chemotherapy or chemoradiotherapy to the two patients and assessed the effects on the primary esophageal cancer and the lymphadenopathy via FDG-PET/CT. As a result, we observed a small change in the hilar and mediastinal lymph nodes despite the complete disappearance of FDG uptake in the primary tumor in both patients (Table 1). These different responses between the primary and lymph node lesions suggest that the lymphadenopathies are very likely non-metastatic lesions, sarcoidosis, or sarcoid reactions.

**Table 1:** Comparison of the SUV max before and after chemotherapy/chemoradiotherapy

		Before	After
Case 1	Primary lesion	4.06	Disappeared
	Bilateral hilar lymph nodes	15.00	8.07
	Paraesophageal lymph nodes	6.94	5.08
Case 2	Primary lesion	23.10	Disappeared
	Paraesophageal lymph nodes	5.45	3.26

FDG-PET/CT appears to be useful for staging, evaluating disease activity, and monitoring treatment responses in patients with sarcoidosis [19]. Therefore, FDG-PET/CT findings should be interpreted with caution. A previous study by Kaira et al. has suggested that using the more tumor-specific tracer L-[3-^18^F]-α-methyltyrosine in an FDG-PET/CT scan may be an effective method to distinguish sarcoidosis from malignancy [20]. Both patients in this study were administered steroids to prevent side effects of chemotherapy. Corticosteroids represent the most common agents used in sarcoidosis therapy, and the current regimen for treatment is 30–40 mg of prednisone daily for 8–12 weeks [21]. Therefore, steroids in chemotherapy might slightly reduce FDG uptake in the mediastinal lymph nodes. These clinical responses and the possible etiology of FDG uptake are thought to be new findings to the literature [11].

In esophageal cancer patients with suspected lymphadenopathies from sarcoidosis or sarcoid-like reactions, monitoring the response to chemotherapy via FDG-PET/CT may be helpful to diagnose whether the lymphadenopathies are benign or metastatic. However, this method may not be applicable when the esophageal cancer does not respond to chemotherapy/chemoradiotherapy. Nevertheless, 84% of patients with esophageal squamous cell carcinoma demonstrate good responses to neoadjuvant chemotherapy on FDG-PET/CT.

The previous case monitored by Bekki et al. was a sarcoid-like reaction. The data of our first case support the suggestions of Bekki et al., and we showed that bilateral FDG accumulation in the hilar lymph nodes was only slightly changed after 15 months. In our second case, we have newly reported that FDG-PET/CT has diagnostic utility in the differential diagnosis of lymphadenopathy, even in patients with sarcoidosis. Therefore, monitoring responses by FDG-PET/CT is useful both for lesions associated with a sarcoid-like reaction and those from systemic sarcoidosis, although the incidence of esophageal cancer complicated by sarcoidosis is rare. Analyses in a large series of esophageal cancer patients with benign and malignant lymphadenopathy are needed to assess the differential diagnostic ability of FDG-PET/CT monitoring.

## Abbreviations

CT: Computed tomography
FDG: [^18^F]-fluorodeoxyglucose
FDG-PET/CT: [^18^F]-fluorodeoxyglucose positron emission tomography–computed tomography
MRI: Magnetic resonance imaging
SUV-max: Maximum standardized uptake value
UICC: Union for International Cancer Control

## References

[1] Kato H, Kimura H, Nakajima M, Sakai M, Sano A, Tanaka N (2008). The additional value of integrated PET/CT over PET in initial lymph node staging of esophageal cancer. Oncol Rep.

[2] Yanagawa M, Tatsumi M, Miyata H, Morii E, Tomiyama N, Watabe T (2012). Evaluation of response to neoadjuvant chemotherapy for esophageal cancer: PET response criteria in solid tumors versus response evaluation criteria in solid tumors. J Nucl Med.

[3] Kato H, Fukuchi M, Miyazaki T, Nakajima M, Tanaka N, Inose T (2007). Prediction of response to definitive chemoradiotherapy in esophageal cancer using positron emission tomography. Anticancer Res.

[4] Kato H, Kuwano H, Nakajima M, Miyazaki T, Yoshikawa M, Masuda N (2002). Usefulness of positron emission tomography for assessing the response of neoadjuvant chemoradiotherapy in patients with esophageal cancer. Am J Surg.

[5] Wahl RL, Jacene H, Kasamon Y, Lodge MA (2009). From RECIST to PERCIST: evolving considerations for PET response criteria in solid tumors. J Nucl Med.

[6] Newman LS, Rose CS, Maier LA (1997). Sarcoidosis. N Engl J Med.

[7] Hirota T, Kaneda M, Iwasa M, Tamaki H (1993). A case report of gastric cancer associated with sarcoid reactions in the regional lymph nodes and liver. Surg Today.

[8] Gregorie HB, Othersen HB, Moore MP (1962). The significance of sarcoid-like lesions in association with malignant neoplasms. Am J Surg.

[9] Gorton G, Linell F (1957). Malignant tumours and sarcoid reactions in regional lymph nodes. Acta Radiol.

[10] Nadel EM, Ackerman LV (1950). Lesions resembling Boeck’s sarcoid in lymph nodes draining an area containing a malignant neoplasm. Am J Clin Pathol.

[11] Bekki Y, Kimura Y, Morita M, Zaitsu Y, Saeki H, Okamoto T (2015). Esophageal cancer associated with bilateral hilar lymphadenopathy caused by sarcoid-like reactions: a report of two cases. Esophagus.

[12] Brincker H (1987). Solid tumors preceding or following sarcoidosis. Med Pediatr Oncol.

[13] Heinle R, Chang C (2014). Diagnostic criteria for sarcoidosis. Autoimmun Rev.

[14] Bassler R, Birke F (1988). Histopathology of tumour associated sarcoid-like stromal reaction in breast cancer. An analysis of 5 cases with immunohistochemical investigations. Virchows Archiv A, Pathol Anat Histopathol.

[15] Llombart A, Escudero JM (1970). The incidence and significance of epithelioid and sarcoid-like cellular reaction in the stromata of malignant tumours. A morphological and experimental study. Eur J Cancer.

[16] Brincker H (1986). Sarcoid reactions in malignant tumours. Cancer Treat Rev.

[17] Parra ER, Canzian M, Saber AM, Coelho RS, Rodrigues FG, Kairalla RA (2004). Pulmonary and mediastinal “sarcoidosis” following surgical resection of cancer. Pathol Res Pract.

[18] Schauer M, Theisen J (2010). The diagnostic challenge of mediastinal sarcoidosis accompanying esophageal cancer. World J Surg Oncol.

[19] Treglia G, Annunziata S, Sobic-Saranovic D, Bertagna F, Caldarella C, Giovanella L (2014). The role of 18F-FDG-PET and PET/CT in patients with sarcoidosis: an updated evidence-based review. Acad Radiol.

[20] Kaira K, Oriuchi N, Otani Y, Yanagitani N, Sunaga N, Hisada T (2007). Diagnostic usefulness of fluorine-18-alpha-methyltyrosine positron emission tomography in combination with 18F-fluorodeoxyglucose in sarcoidosis patients. Chest.

[21] Dastoori M, Fedele S, Leao JC, Porter SR (2013). Sarcoidosis - a clinically orientated review. J Oral pathol Med.

